# Supplementary material for: The interactome of intact mitochondria by cross-linking mass spectrometry provides evidence for coexisting respiratory supercomplexes
Source: Mol Cell Proteomics. 2017 Dec 8;17(2):216–32. doi: 10.1074/mcp.RA117.000470 (PMC5795388; doi:10.1074/mcp.RA117.000470)
Supplement: Supplemental Data [file supp_RA117.000470_133922_0_supp_23978_fzffwf.zip › spectra_annotation/mito_spectra_annotation/122-1-4-1-12-1.pdf]

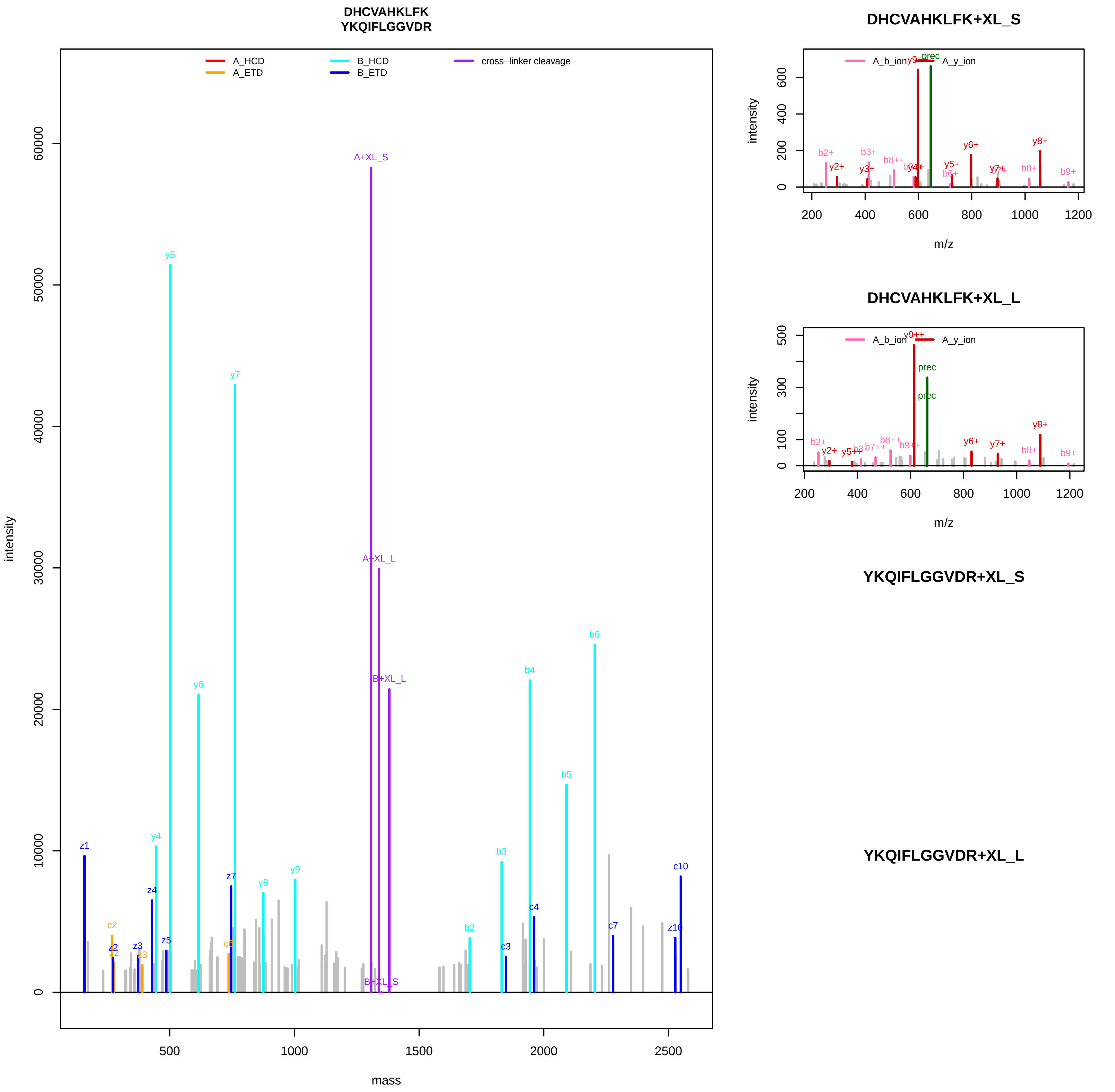

DHCVAHKLFK
YKQIFLGGVDR
A_HCD
A_ETD
B_HCD
B_ETD
cross−linker cleavage
A+XL_S
A+XL_L
B+XL_L
B+XL_S
y4
y5
y6
y7
y8
y9
z1
z2
z3
z4
z5
z7
z10
c2
c3
c4
c6
c7
c10
b2
b3
b4
b5
b6
intensity
mass
DHCVAHKLFK+XL_S
DHCVAHKLFK+XL_L
A_b_ion
A_y_ion
prec
m/z
YKQIFLGGVDR+XL_S
YKQIFLGGVDR+XL_L
